# Developing a clinical decision support system software prototype that assists in the management of patients with self-harm in the emergency department: protocol of the PERMANENS project

**DOI:** 10.1186/s12888-024-05659-6

**Published:** 2024-03-20

**Authors:** Philippe Mortier, Franco Amigo, Madhav Bhargav, Susana Conde, Montse Ferrer, Oskar Flygare, Busenur Kizilaslan, Laura Latorre Moreno, Angela Leis, Miguel Angel Mayer, Víctor Pérez-Sola, Ana Portillo-Van Diest, Juan Manuel Ramírez-Anguita, Ferran Sanz, Gemma Vilagut, Jordi Alonso, Lars Mehlum, Ella Arensman, Johan Bjureberg, Manuel Pastor, Ping Qin

**Affiliations:** 1 Hospital del Mar Research Institute, Barcelona Biomedical Research Park (PRBB), Carrer Doctor Aiguader, 88, 08003 Barcelona, Spain; 2 CIBER of Epidemiology and Public Health, Carlos III Health Institute (CIBERESP, ISCIII), Madrid, Spain; 3 School of Public Health & National Suicide Research Foundation, University College Cork, Cork, Ireland; 4 Centre for Psychiatry Research, Department of Clinical Neuroscience, Karolinska Institutet, & Stockholm Health Care Services, Region Stockholm, Sweden; 5 National Centre for Suicide Research and Prevention, Institute of Clinical Medicine, University of Oslo, Oslo, Norway; 6 Research Programme on Biomedical Informatics (GRIB), Hospital del Mar Research Institute, Barcelona, Spain; 7 Department of Medicine and Life Sciences, Universitat Pompeu Fabra, Barcelona, Spain; 8 Neuropsychiatry and Drug Addiction Institute, Barcelona MAR Health Park Consortium PSMAR, Barcelona, Spain; 9 CIBER of Mental Health and Carlos III Health Institute (CIBERSAM, ISCIII), Madrid, Spain; 10 Department of Paediatrics, Obstetrics and Gynaecology and Preventive Medicine and Public Health Department, Universitat Autònoma de Barcelona (UAB), Barcelona, Spain; 11 National Bioinformatics Institute - ELIXIR-ES (IMPaCT-Data-ISCIII), Barcelona, Spain

**Keywords:** Suicide, Intentional self-harm, Hospital Emergency Service, Clinical decision support system, Machine learning, Risk Assessment, Routinely Collected Health data, Knowledge bases user-Centred Design.

## Abstract

**Background:**

Self-harm presents a significant public health challenge. Emergency departments (EDs) are crucial healthcare settings in managing self-harm, but clinician uncertainty in risk assessment may contribute to ineffective care. Clinical Decision Support Systems (CDSSs) show promise in enhancing care processes, but their effective implementation in self-harm management remains unexplored.

**Methods:**

PERMANENS comprises a combination of methodologies and study designs aimed at developing a CDSS prototype that assists clinicians in the personalized assessment and management of ED patients presenting with self-harm. Ensemble prediction models will be constructed by applying machine learning techniques on electronic registry data from four sites, i.e., Catalonia (Spain), Ireland, Norway, and Sweden. These models will predict key adverse outcomes including self-harm repetition, suicide, premature death, and lack of post-discharge care. Available registry data include routinely collected electronic health record data, mortality data, and administrative data, and will be harmonized using the OMOP Common Data Model, ensuring consistency in terminologies, vocabularies and coding schemes. A clinical knowledge base of effective suicide prevention interventions will be developed rooted in a systematic review of clinical practice guidelines, including quality assessment of guidelines using the AGREE II tool. The CDSS software prototype will include a backend that integrates the prediction models and the clinical knowledge base to enable accurate patient risk stratification and subsequent intervention allocation. The CDSS frontend will enable personalized risk assessment and will provide tailored treatment plans, following a tiered evidence-based approach. Implementation research will ensure the CDSS’ practical functionality and feasibility, and will include periodic meetings with user-advisory groups, mixed-methods research to identify currently unmet needs in self-harm risk assessment, and small-scale usability testing of the CDSS prototype software.

**Discussion:**

Through the development of the proposed CDSS software prototype, PERMANENS aims to standardize care, enhance clinician confidence, improve patient satisfaction, and increase treatment compliance. The routine integration of CDSS for self-harm risk assessment within healthcare systems holds significant potential in effectively reducing suicide mortality rates by facilitating personalized and timely delivery of effective interventions on a large scale for individuals at risk of suicide.

## Background

Self-harm presents a significant, yet preventable, public health issue, affecting a minimum of 14.6 million individuals globally each year [1]. Individuals with self-harm have elevated risk for repetition of self-harm [2–4] and premature death by suicide [2, 3] and other causes [5, 6]. Over 700,000 people die by suicide each year [7], representing an estimated loss of 34.6 million years of life [8]. Furthermore, up to 135 individuals may be affected or bereaved by each suicide [9]. In Europe, suicide rates are notably high (12.8/100,000 in 2019), yet understanding the prevalence and determinants of self-harm and suicidal behaviour in Europe remains a challenge. This is due to the absence of comprehensive surveillance efforts and the considerable differences in definitions and methodologies used across studies and countries [10].

Between 49% and 60% of individuals who die by suicide had visited an emergency department (ED) in the year before their death [11–13]. EDs play a pivotal role in the care for people with self-harm [14, 15] as they often represent the first medical contact after self-harm and can offer specialized risk assessment and treatment referral. Several effective interventions for preventing self-harm exist [16]. These include brief interventions such as care coordination, development of safety plans, offering brief follow-up contacts, and targeted therapeutic interventions. When administered during a single encounter with patients at risk of suicide, such as those who have self-harmed and visited the ED, these interventions may not only reduce subsequent suicide attempts but also improve the likelihood of connecting individuals to follow-up care [17]. It is however unclear which interventions are most effective for particular suicide risk profiles, underlining the need for studies to support precision medicine approaches [18–20].

A longstanding concern in mental healthcare is the low uptake of proposed treatment by patients that present with self-harm at the ED. According to a recent meta-analysis encompassing 131 distinct studies, roughly two-thirds of these patients receive referrals for either in- or outpatient care, with considerable variability in the factors influencing the allocation of treatment. Importantly, only about one in five effectively receive the recommended treatment [21] and non-attendance among those referred is a strong predictor for subsequent death by suicide [22].

One potential explanation for this low treatment uptake may lie in the varying quality of care provided at the ED to individuals who self-harm. Quality of care as it is perceived by patients ranges from genuine and empathetic healthcare interactions that empower patients to explore reasons for their distress, leading to more effective care, to superficial, disconnected contacts. The latter can include rushed, formulaic risk assessments, a tendency to minimize patients’ distress, and perceived lack of trust, all of which impair willingness to engage in and comply with treatment [23–28]. Moreover, there is often a lack of involving and educating informal caregivers when treating people with self-harm [29, 30]. Crucially, unsupportive care has shown to be significantly associated with repeat self-harm [31]. Related to this, so-called frequent self-harm repeaters have been identified, i.e., small proportions of patients with self-injury (< 1%) that are frequent users of (emergency) services but with low adherence and response to mental health treatment [32] which contribute to the high societal costs related to self-harm.

Sub-optimal quality of care for individuals who self-harm could stem, in part, from healthcare providers experiencing feelings of insecurity or uncertainty, often related to stigma and lack of specialist training. Risk detection and intervention efforts at the ED are complicated because self-harm represent difficult-to-predict complex behaviour [20, 33, 34]. Unassisted clinician assessments are insufficient to accurately identify patients at highest risk for repeated self-harm and suicide [35, 36]. Clinicians are, therefore, prone to heuristic-based decision making (i.e., using simplified mental shortcuts or rules of thumb) by linking a limited (and often arbitrary) set of risk factors directly to suicide potential [37]. Addressing the self-perceived lack of training among ED clinicians [38–40] may mitigate this problem. However, even among trained mental healthcare professionals, there is high variability in clinical judgement regarding suicide risk assessment [41, 42]. Psychiatrists report that suicide risk assessments are often based on semi-intuition or gut feeling, leading to concerns of being unprofessional and to feelings of loneliness and insecurity [43]. This, in turn, may lead to ineffective clinical decision-making, poor patient experience, and adverse outcomes such as involuntary admissions based on uncertainty and preventable deaths by suicide. Relying on the use of standardized risk assessment scales does not solve this issue as these scales have proven to be inaccurate [44] and may lead to the false impression that risk management is based on objective evidence [30, 43].

Clinical Decision Support Systems (CDSS) have shown the potential to enhance care processes in diverse clinical settings by boosting the precision of risk assessments and refining evidence-based treatment allocation based on risk stratification [45, 46]. Increasingly, CDSSs are being developed with the capability to conduct assessments based on information otherwise unobtainable by humans, using machine learning-based prediction models that allow for considering the complex interactions over time between a high number of patient characteristics, derived from large sets of electronic healthcare registry data. In suicide research, it has been shown that machine learning-based suicide risk predictions outperform all widely researched theories of suicide [47], opening the possibility to stratify patients with self-harm according to risk for adverse outcomes, such as death by suicide, and provide tailored intervention preventions. Despite their clear usefulness in other clinical domains [48–51], evidence of effective implementation of these techniques into clinically useful prediction tools for self-harm and related adverse outcomes is lacking. This may be due to the absence of a user-oriented personalised approach, i.e., the failure to actively involve both patients and clinicians in the development of this kind of software tools [52]. Qualitative studies reveal healthcare providers’ interest in machine learning–based risk prediction systems, highlighting concerns like liability, alert fatigue, and increased healthcare system demand [53]. In addition, more research is needed on how to integrate CDSS tools to improve suicide prevention training frameworks that take into account both risk assessment and recovery-oriented approaches [54].

### Summary of relevant previous studies

While statistical risk prediction models have shown some promise in improving suicide risk prediction accuracy [55–58] and cost-effectiveness [59], their application in clinical settings remains limited. The U.S. Veterans’ Health Administration utilized a statistical model in the REACH VET program to identify at-risk veteran patients and link them with care [60, 61]. Jaspr Health introduced a tablet-based digital assistant, featuring an AI-driven chatbot aiding ED patients with suicide risk and establishing evidence-based discharge plans, proving both feasible and acceptable [62]. e-Connect is a digital CDSS that improves suicide risk identification and referral among youth in the justice system by probation officers [63]. Finally, the OxRisk project [64] developed tools that provide suicide risk probability scores for individuals presenting with self-harm (OxSATS, [65]) and severe mental illness (OxMIS, [66]). To the best of our knowledge, no prior study has developed a CDSS specifically designed to facilitate personalized assessment and management for unselected populations of patients presenting with self-harm at the emergency department, including risk estimation for various relevant adverse outcomes, such as self-harm repetition and death by suicide. Additionally, prior research has not integrated implementation research to ensure the CDSS’s patient focus, practical value, and usability in real-world healthcare settings.

### Aims

Here, we outline the research protocol of the PERMANENS project (www.permanens.eu). PERMANENS is a European research project that brings together expertise in clinical mental health research, public health, biostatistics, and biomedical informatics to develop a CDSS software prototype that can assist clinicians in the personalized detection, assessment and management of risk for key adverse clinical outcomes among ED patients presenting with self-harm. The key adverse clinical outcomes that we aim to tackle in this project include repeat self-harm, self-harm method lethality escalation, death by suicide, premature death, and not receiving mental health treatment following discharge from ED. Trained on evidence accumulated in clinical settings and based on the patient’s particular clinical history, the CDSS will provide the clinician with personalized risk profiles for these adverse outcomes, and propose an evidence-based treatment plan, tailored to the patient’s specific risk profile.

The proposed CDSS will introduce a structured professional judgement approach [67] for self-harm management at the ED– a systematic approach for clinicians to evaluate risk factors, consider historical data, and weigh various elements in the decision making process in a comprehensive way. Importantly, this implies that the proposed CDSS will not be a mere actuarial or statistical instrument, but instead focuses on guiding the clinician-patient interaction to ensure that all relevant areas of clinical risk are evaluated and that adequate treatment is being offered in a highly personalised way. This approach is expected to improve standardization of care as well as clinicians’ confidence in their judgement by guiding the creation of a clinical formulation and a risk management plan. This approach will also enable clinicians to dedicate mental resources to conducting more empathetic and collaborative assessments, acknowledging specific patients’ needs and values, hereby further improving patient satisfaction and treatment adherence.

## Methods

### Study design

**Fig. 1:**
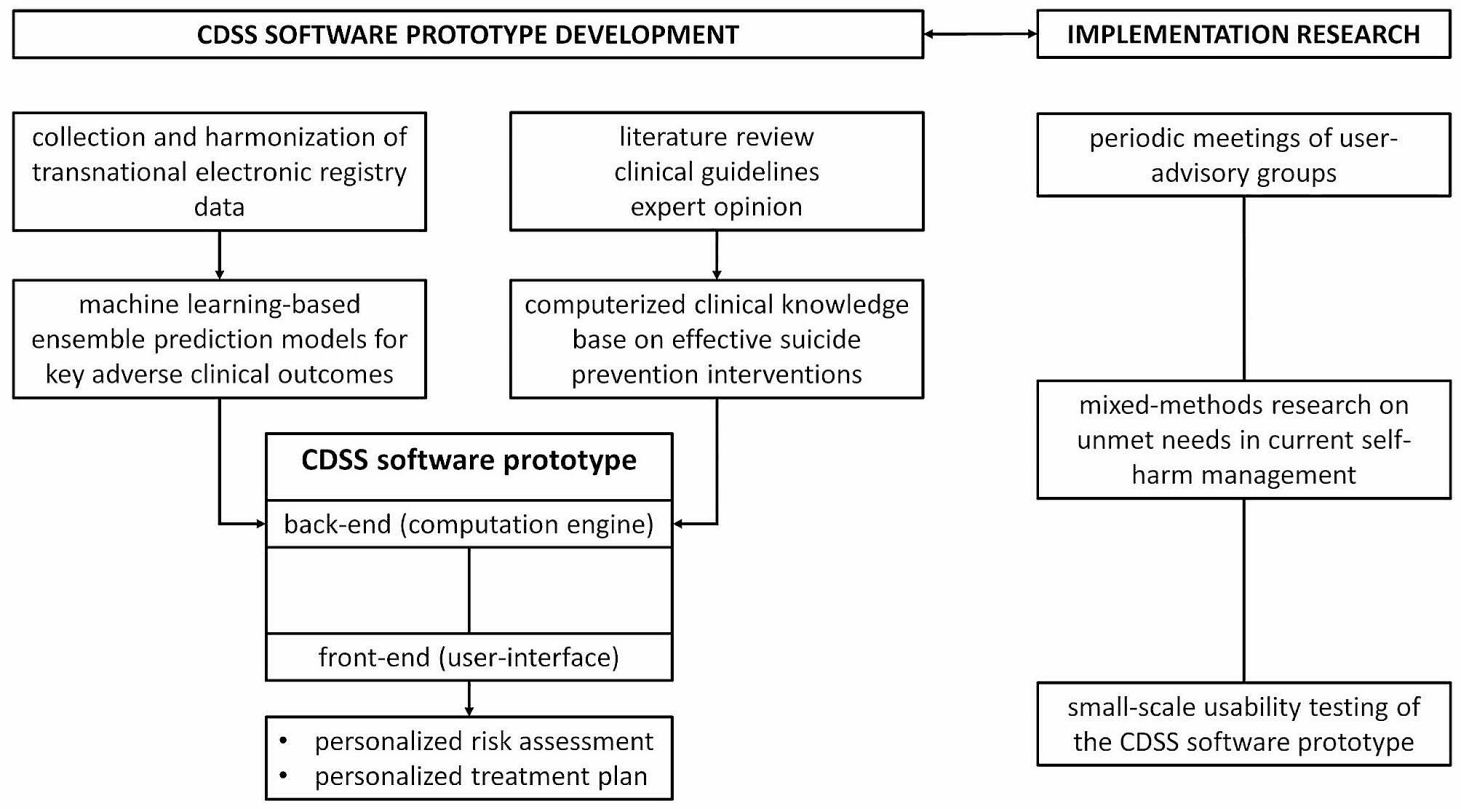
Overview of PERMANENS CDSS software prototype development and related research activities

The PERMANENS project consists of a combination of methodologies and study designs oriented to the development of a CDSS software prototype, as briefly summarized in Fig. 1. In summary, the CDSS software backend (i.e., the part of the software that allows it to operate but cannot be accessed by the user) will consist of two main components: (1) a series of prediction models for key adverse clinical outcomes, obtained by machine learning-based techniques from transnationally harmonized health record data; and (2) a computerized clinical knowledge base on effective suicide prevention interventions, obtained through a systematic review of the literature, including clinical guidelines. The proposed CDSS software frontend (i.e., the part that interacts with the end-user) will exploit the backend resources to facilitate personalized risk assessment and the delivery of a personalized treatment plan, tailored to the patient’s specific risk profile and needs. User-oriented implementation research will consist of periodic user-advisory group meetings throughout the project, mixed-methods research on currently unmet needs in self-harm management, and small-scale usability testing of the CDSS prototype. Below we describe each of the research activities in more detail.

### Collection of transnationally harmonized electronic registry data

The data used for the development of prediction models for key clinical adverse outcomes consists of individual-level quantitative structured data from existing electronic registries from four separate sites, i.e., three countries (Ireland, Norway, Sweden) and one region (Catalonia, Spain; see Table 1). Types of registry data available at all sites include routinely collected electronic health record (EHR) data, mortality data, and administrative data. Routinely collected EHR data is coded using the International Classification of Diseases-9th revision-Clinical Modification (ICD-9-CM) and ICD-10-CM disease classification systems. This data will be used to identify ED visits of patients presenting with self-harm, including information on repeat self-harm, method escalation (i.e., transitioning to the use of more lethal self-harm methods), and the patients’ clinical history, encompassing mental and substance use disorders, and pertinent somatic conditions. Mortality data is coded using the International Classification of Disease 10 (ICD-10) coding system and will be used to extract information on death by suicide and premature mortality. Administrative data will be used to extract information on sex, age, and socio-economic status. On some sites, data will be complemented with pharmaceutical registry data (Catalonia, Spain, and in Sweden) and/or with specific self-harm case registry data (i.e., the National Self-Harm Registry in Ireland [68]; the Suicide Risk Code Registry in Catalonia, Spain [69]).

**Table 1:** Overview of electronic registry data used in the PERMANENS project

	Catalonia (Spain)	Ireland	Norway	Sweden	Purpose of data
Routinely collected healthcare registry data	Available	Available	Available	Available	Defining cohort of ED patients presenting with self-harm Defining outcome variables: repeat self-harm, method escalation Defining predictor variables: patients’ clinical history
Mortality data	Available	Available	Available	Available	Defining outcome variables: death by suicide, premature death
Administrative data	Available	Available	Available	Available	Defining predictor variables: age, sex, socio-economic status
Pharmaceutical registry data	Available	Not Available	Not Available	Available	Defining predictor variables: pharmaceutical drugs prescribed and/or dispensed
Self-Harm case registry data	Available	Available	Not Available	Not Available	Defining cohort of ED patients with self-harm Defining outcome variables: repeat self-harm, method escalation
Sources of electronic registry data	Data Analytics Program for Health Research and Innovation (PADRIS) of the Agency for Health Quality and Assessment of Catalonia (AQuAS)	National Self-Harm Registry Ireland, Hospital In-Patient Enquiry	Norwegian Patient Registry, Norwegian Cause-of-Death Register, Statistics Norway’s Events Database, Norwegian Central Population Registry	Swedish National Patient Registry, Swedish Cause-of-Death Register, Swedish Total Population Register, Swedish Longitudinal Integration Database for Health Insurance and Labour Market Studies	/

The Observational Medical Outcomes Partnership (OMOP) Common Data Model (CDM) [70] developed by the Observational Health Data Sciences and Informatics (OHDSI) [71] will be used in order to transform and harmonise all registry data from the four sites into a common data format using standard terminologies, vocabularies and coding schemes.

### Machine learning-based prediction models for key adverse clinical outcomes

Next, to facilitate analysis of registry data across sites, we will use a federated data analysis methodology [72, 73]. This methodology involves within-country analyses of individual-level data, producing local aggregated results, saved as query objects (i.e., analysis result that has been transformed or aggregated without any individual-level data remaining, e.g., predictive models or descriptive tables). These results are then exported and centralized for further aggregated analysis at a transnational level, eliminating the need for centralized data storage or cross-border access to local individual-level data. This approach ensures compliance with national and European personal data privacy regulations.

In order to prepare the data for subsequent predictive modelling, we will delineate a patient cohort, operationalize the key adverse clinical outcomes, define the predictor variables for these outcomes, and identify clinically relevant subgroups of self-harm patients. The cohort will consist of patients presenting with self-harm at the ED, and index visits will consist of the first ED visit for self-harm during a predefined period of time. Key adverse clinical outcomes following index ED visit will include repeat self-harm (i.e., having subsequent ED visits for self-harm after the index visit), self-harm method escalation, death by suicide, premature death (e.g., death by any cause before age 70), and not receiving any post-discharge treatment (i.e., not having any inpatient or outpatient psychiatric care registered in the period following ED discharge). Building on previous evidence [74, 75], data-driven diagnostic algorithms will be developed to facilitate accurate identification of self-harm incidents in the registry data.

Predictor variables (features) for the key adverse clinical outcomes will reflect patients’ available clinical information prior to index ED visit, including self-harm, mental and substance use disorders, somatic conditions, and receipt of inpatient or outpatient mental healthcare. Composite predictor variables reflecting mental and/or somatic comorbidity will also be created, as well as separate predictor variables reflecting different retrospective time periods with respect to the index visit (e.g., past 30, 90, 180, 365 days). Clinically relevant subgroups of self-harm patients will be identified (e.g., based on gender, age, socio-economic status, mental disorder category) and the convenience of developing separate prediction models for them will be evaluated. These subgroups will be identified based on literature review, expert opinion as well as data analysis, ranging from simple descriptive analysis to unsupervised machine learning approaches.

Next, we will develop and validate a series of clinically interpretable prediction models which will enable the accurate stratification of patients according to risk for the key adverse clinical outcomes. We will develop machine learning-based models, including ensembles of decision trees (random forests and gradient boosting methods) and deep learning models if the size and depth of the data justify its use. To address class imbalance, pseudo-sampling of cases and under-sampling of controls will be considered. To enable clinical interpretability of prediction models [76], a set of most important predictors will be identified for each model (e.g., using SHapley Additive exPlanations [SHAP] metrics) in terms of their contribution to the overall prediction accuracy. Also, clinical interpretability will be one of the criteria (alongside accuracy metrics such as sensitivity and specificity) to choose the final predictive model to be deployed.

Prediction models will first be developed separately at the local (i.e., within-country) level. These local models will be stored as query objects to allow them to be shared across sites without the need of remotely accessing the original individual-level data, in compliance with national and European data security and privacy regulations. These models will be used to build an ensemble model, which can be exploited for: (1) externally cross-validating each of the models in each of the other countries’ data, substantially increasing the models’ robustness; (2) investigating key differences across countries with respect to the importance of the clinical variables; (3) identifying predictors that are in need for assessment in specific countries; and (4) pooling together the predictive power of each predictor variable across countries using meta-analytic techniques to obtain estimates of prediction accuracy of the global model on the conjoint set of transnational data.

Potential prediction bias in the prediction models with regard to relevant variables (e.g., sex, age group, socio-economic status) will be investigated and eliminated by systematically including these variables as fixed covariates throughout model development as well as by developing separate models stratified by different values of these variables, whenever it will be considered necessary.

### Clinical knowledge base on effective suicide prevention interventions

A systematic review will be conducted to develop a knowledge base for evidence-based assessment and treatment options for individuals with self-harm or suicidal behaviour. This will consist of a systematic literature search of available clinical practice guidelines (CPGs) for patients aged 18 or older presenting in any healthcare setting. Inclusion of CPGs will not restricted by language and will be translated to English prior to full-text review. CPGs will be identified from Ovid MEDLINE, Ovid MEDLINE In-Process & Other Non-Indexed Citations, EMBASE, Web of Science, PsycINFO, and the Cochrane Library. Gray literature will be searched using Guidelines International Network (GIN), Trip Medical Database, and the National Institute for Health and Care Excellence in the United Kingdom (NICE). The search will be complemented by sourcing guidelines documents from researchers in the International Academy of Suicide Research. The AGREE II tool [77–79] will be used to assess the quality of included CPGs, considering the following domains: Scope and purpose, Stakeholder involvement, Rigour of development, Clarity of presentation, Applicability, and Editorial independence. Findings from the systematic review will include quality of the reviewed CPGs, recommendation matrices for outcomes included in high-quality CPGs, the levels of evidence underpinning recommendations, comparative treatment preferences, and emerging themes in the material. The systematic review protocol is registered with PROSPERO (CRD42023488333).

### CDSS prototype software development

Development of the software backend (see Fig. 1) will consist of implementing the machine learning-based prediction models and the computerized clinical knowledge base on effective suicide prevention interventions. Using this system, and based on the data of an individual patient, the system will assign a predicted risk score for the key adverse clinical outcomes and provide suggestions for treatment interventions. The software will work as a web-service, exposing a RESTful application programming interface (API) through which it will interchange input and output data with the front-end.

Development of the software frontend will consist of designing a graphical user interface (GUI) that will ensure intuitive and user-friendly human-system interactions. The CDSS will enable for clinicians to apply a tiered evidence-based approach in the clinical management of self-harm at the ED. The personalised CDSS main outputs will consist of: (1) risk scores (0-100%) and visualization, including the risk of key adverse clinical outcomes, and specifying the most important predictors; (2) recommendations of risk factors in need for assessment to further optimise the ongoing risk evaluation; and (3) a personalised treatment plan aimed at delivery of effective prevention interventions and at ensuring continuity of care. It is important to stress that final assignment of treatment will not be entirely data-driven (i.e., rely on predicted risk scores) but will also be based on expert opinion on which treatment is most indicated considering relevant clinical variables such as age, gender, and mental disorder diagnosis. Risk for intrusive interventions provoked by false-positive model predictions will be prevented by the fact that the final decision regarding treatment and individual rights restriction always lies with the end-user (clinician and/or patient) and never the CDSS. Nevertheless, intensity of the proposed interventions will always depend on the level of certainty of the prediction, and proportional to the risk detected.

We will also develop an format for a transferable personal healthcare record, co-created with patients, and fully acknowledging them as end-users and managers of their data. Such healthcare record format could consist of (but is not limited to) a comprehensive summary of a patient’s risk factors, past healthcare use trajectories, and individualised treatment plans. The final format of the personal healthcare record will consist of a digital portable file (outputted by the CDSS software) that the patient could share with other involved healthcare providers after the risk assessment. The final goal of the transferable personal healthcare record is to improve continuity of care, by enabling more effective communication between patients’ different healthcare providers.

Evaluation of software development will consist of both technical validation (no bugs, adherence to specifications) and functional validation (verifying whether the software meets all users’ real needs). Importantly, we aim for the personalised CDSS to be used as a stand-alone application, not depending on the connection with electronic healthcare systems for its functioning. This means that the input data can consist entirely of user-entered data, i.e., clinicians providing all necessary input data manually through the software GUI. Although this will add user burden at this stage of CDSS development, this is necessary to provide a feasible and flexible software that can also be tested and fine-tuned in healthcare settings with poor system integration [46]. To lower user burden in terms of data that needs to be entered manually, we will test, in a pilot study, the use of prediction models based on sets of predictors with highest predictive accuracy (and minimal loss of overall model prediction accuracy), and explore the implementation of multistage assessments, first prioritizing models with high sensitivity, and subsequently using models with optimum sensitivity and specificity trade-off. Evidently, full integration of the personalised CDSS into electronic healthcare record systems will be desirable as a future step to optimise the integration of the CDSS into the clinical workflow.

### Implementation research with end-users

We will organize periodic meetings with user-advisory groups (UAG) to obtain the necessary knowledge and insights throughout the project, and to ensure commitment from local stakeholders for adoption and future implementation of the CDSS. UAG members will consist of people with personal lived experience of ED visits for self-harm and their caregivers, members of advocacy or patient groups representing persons with lived experience, and ED mental healthcare professionals. At least two meetings will be held yearly at each site, including 4–8 participants in each UAG. To standardize procedures across the four sites, meetings will be held in parallel around the same time of year and using a common meeting agenda. The meeting format will be online group meeting, but the option of in-person personal interviews will be actively offered to people with lived experience, in case they prefer this format due to perceived privacy issues. Moderators for the UAG will consist of research team members with sufficient experience and/or training to handle the sensitive nature of the discussed topics. Recruitment for the UAG will be by personal invitation and/or snowball sampling. Participant information leaflets will be developed for that purpose. All participants will be aged 18 or more. The end-deliverable format of the UAG meetings will be a detailed summary report, including a synthesis of findings and results across the four sites.

Using a mixed-methods research design, we will investigate currently unmet needs in self-harm risk assessment at the ED to ensure that the proposed CDSS’ objectives and methodology maximally address users’ needs. Mixed-methods research will consist of focus groups and web-based surveys. Focus groups will be held at the Ireland and Spain sites only. Recruitment methods for the focus groups, type of participants, moderators, and meeting format will be identical as in the UAG meetings. A predefined list of relevant topics (topic guide) will serve as a guide for the focus groups. A gender- and age-tailored recruitment approach will be used to guarantee representative participation. All participants will be aged 18 or more. Audio-recorded group sessions will be transcribed and subjected to thematic and content analysis. Short web-based surveys implemented using Qualtrics will be used to assess currently unmet needs in suicide risk assessment among ED staff members, Clinical Nurse Specialists, Psychiatric Nurses, Crisis Nurses, Psychiatry residents and registrars, and Clinical Psychologists working in the hospital setting. Snowball sampling methods will be applied for the dissemination of the survey which will be conducted via relevant national networks and professional organizations.

We will conduct a small-scale pilot study for usability testing and validation of the CDSS prototype software with people with personal lived experience of ED visits for self-harm and with ED mental healthcare professionals. Clinician-patient dyads will be established for this purpose, in safe clinical settings (e.g., the emergency or psychiatry department) but outside of routine clinical healthcare. Sample size will be determined through the principle of saturation. This will facilitate an iterative evaluation process that will be conducted using an established methodological framework, providing feedback loops to ongoing tasks in software development. Usability, feasibility, and acceptability of the CDSS will be assessed using well-validated quantitative instruments (e.g., the System Usability Scale) and through qualitative interviews, which will be based on a thorough revision of the literature. Audio-recorded interviews will be transcribed and subjected to thematic and content analysis. By consulting regional and national regulatory authorities, we will start exploring the future feasibility of implementing the CDSS on a larger scale for further clinical validation and implementation.

## Discussion

In this paper, we have presented the protocol of the PERMANENS project, a comprehensive European research initiative focused on the development of a Clinical Decision Support System (CDSS) software prototype. The objective of this prototype is to aid clinicians in the personalized detection, assessment, and management of critical adverse clinical outcomes in emergency department patients presenting with self-harm.

The decision to design a project aimed at developing a CDSS that improves self-harm management at the ED was made without direct patient consultation. However, patient involvement will be maximized throughout all PERMANENS project phases, starting from the outset and continuing across all outlined research activities. Patient involvement will be guaranteed through the implementation research with CDSS end-users to ensure that: (1) the research and in particular the CDSS is influenced by principals of citizenship, accountability, and transparency; (2) to ensure that research is acceptable, accessible, sensitive, and representative of the perspectives of people with lived experience and those involved in providing (mental) health services; (3) to facilitate open and collaborative dialogue between people with lived experience and the research team throughout the development of the CDSS; and (4) to complement the team’s knowledge regarding topics relating to suicide and self-harm.

Dissemination of the PERMANENS project will be conducted through the project’s website (www.permanens.eu); through open-access scientific publications, conferences, workshops and webinars. We will also develop communication strategies (co-created) towards the general public and patients with the focus on creating awareness of the need for a personalised medicine approach in suicide risk assessment. Moreover, we will provide public health policy makers with suggestions of revision in suicide prevention frameworks; and produce an updated clinical guideline for suicide risk assessment, including an online training manual for the clinical use of the CDSS.

Noteworthy aspects of the PERMANENS project include the creation of machine learning-based ensemble prediction models utilizing registry data from diverse European countries, the inclusion of a range of clinically relevant adverse outcomes within the prediction models, and the inclusion of implementation research, actively involving individuals with lived experience and healthcare professionals. This engagement ensures the tailoring of the CDSS software prototype to meet clinical needs, emphasizing its patient focus, practical value, and usability in real-world healthcare settings. Routine implementation of CDSS for self-harm risk assessment in healthcare systems has high potential in effectively reducing suicide mortality in the population, by enabling personalised and timely delivery of effective treatment at large scale among individuals with suicide risk.

## Data Availability

No datasets were generated or analysed during the current study.

## Abbreviations

ED: Emergency Department
CPGs: Clinical Practice Guidelines
ICD-9-CM: International Classification of Diseases-9th revision-Clinical Modification
ICD-10-CM: International Classification of Diseases-10th revision-Clinical Modification
OHDSI: Observational Health Data Sciences and Informatics
OMOP: Observational Medical Outcomes Partnership
CDSS: Clinical Decision Support Systems
API: Application Programming Interface
GUI: Graphical User Interface
UAG: User-Advisory Groups
CRD: Centre for Reviews and Dissemination
PROSPERO: International Prospective Register of Systematic Reviews
RESTful: Representational State Transfer
SHAP: SHapley Additive exPlanations
GIN: Guidelines International Network
NICE: National Institute for Health and Care Excellence
